# Simple Step Add-in to Improve Patient Safety: A Technical Note

**DOI:** 10.7759/cureus.6592

**Published:** 2020-01-07

**Authors:** Elham A Elgabaly, Sattar Alshryda, Haidar Alfuqaha, Ling Hong Lee

**Affiliations:** 1 Pediatric Radiology, Al Jalila Children Specialty Hospital, Dubai, ARE; 2 Pediatric Orthopedics, Al Jalila Children Specialty Hospital, Dubai, ARE; 3 Pediatric Orthopedics, Alkhalidi Hospital, Amman, JOR; 4 Pediatric Orthopedics, Sunderland Royal Hospital, Sunderland, GBR

**Keywords:** human errors, operating room, safety, ampules, medical quality

## Abstract

Human factors and systems factors can affect surgical performance, including the operating room (OR) environment, teamwork and communication, technology and equipment, tasks and workload factors, and organizational variables.

Patient safety is a new healthcare discipline that emphasizes the reporting, analysis, and prevention of medical errors that often lead to adverse healthcare events.

We are highlighting a potential error and hazardous situation, which may occur due to the difficulty in reading the embossed letters of some ampoules because of the typeface of these ampoules. This problem is particularly important in the ORs, which require special sterile conditions.

We are adding a simple step to help in the differentiation between plastic, embossed ampoules. This simple and easy-to-do step makes it possible for accurate and correct identification, without jeopardizing the safety of the patients and health care professionals.

## Introduction

Historically, surgical outcomes have been attributed primarily to the technical skills of the surgeon and the medical condition and comorbidities of the patients. Recently, many studies have reported that the surgical outcome is not only affected by these two factors and that other factors can contribute to the outcome of the surgery, such as the work system factors, the human factor, the operating room (OR) environment, teamwork, and communication [1].

While the precise incidence and epidemiology of medical mistakes still elicit debate, all can agree that human errors are inevitable in any endeavor. Errors typically have little to no consequence and often go unnoticed, but, occasionally, they translate into an adverse event. In the medical setting, this may be reflected in prolonged hospital stays, morbidities, or mortalities [2].

One potential mistake is to use the wrong medication because of similar ampoule shapes and colors. A wide range of plastic, embossed ampoules, including those containing local anesthetics, potassium, and saline, have poor contrast between the typeface and background, presenting a challenge for accurate and easy identification. Here, we propose a simple step to help in the differentiation between these ampoules.

## Technical report

Medicine labels must be clear, legible, and easily read to prevent mistakes [3]. Local anesthetics are commonly used agents in surgery and radiological procedures. They are usually administered in a sterile or aseptic environment, with the scrub nurse holding the ampoule for the surgeon to crosscheck its expiry date and content.

As a consequence of poor visibility, the ampoule is typically held close to the surgeon. What follows thereafter is not uncommon: the scrub nurse will fidget with the ampoule to enable the label to be read, or the surgeon will want to hold the ampoule, which is usually not sterile. This is a potentially risky situation where it presents the opportunity for a sharps injury or infection.

**Figure 1:**
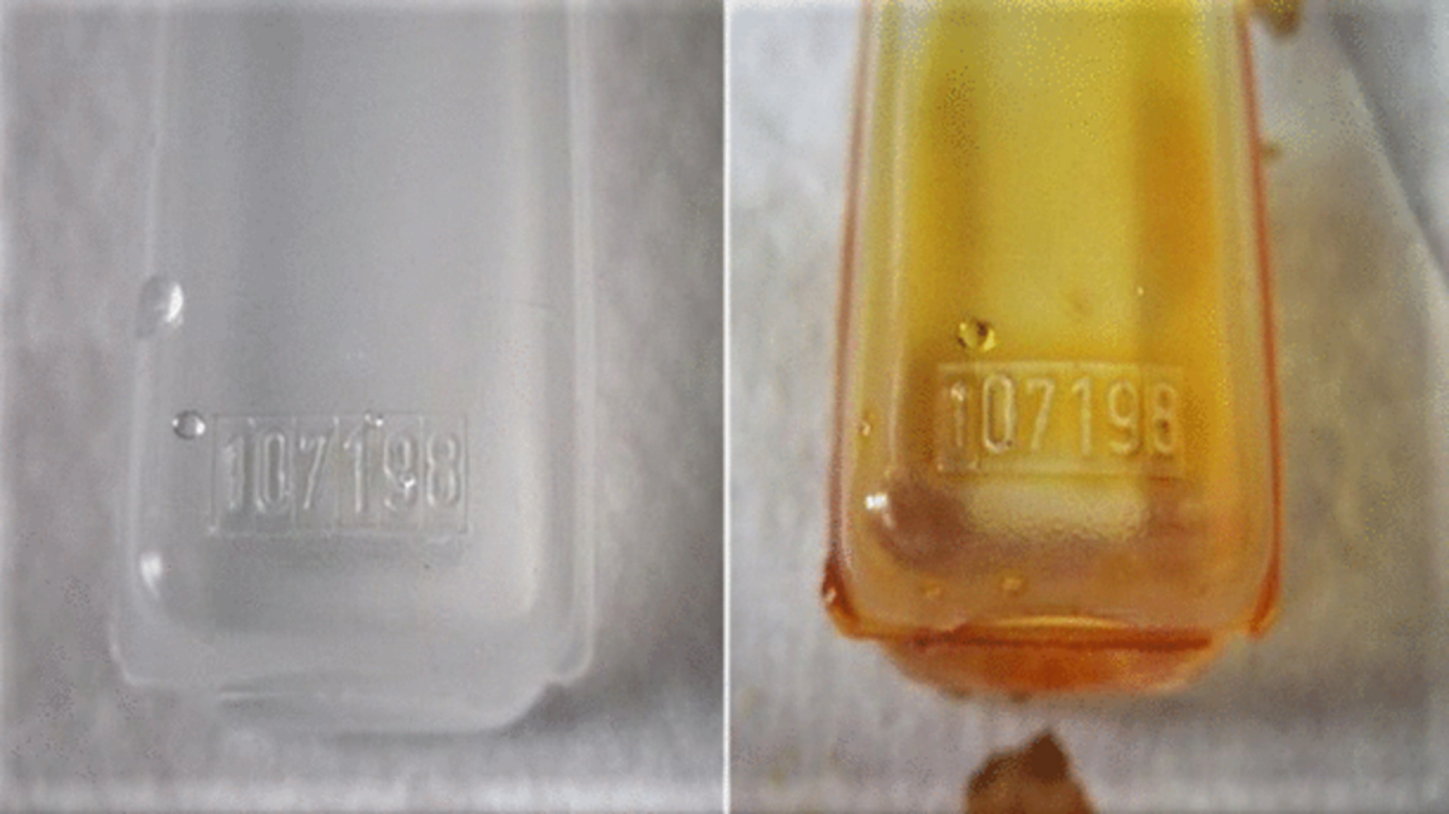
Photograph showing an ampoule before and after Betadine dip

We find that dipping the clear plastic, embossed ampoule in a readily available povidone-iodine antiseptic solution at the start of the procedure, or when needed, improves contrast and reading (Figure 1) and renders the vial sterile. This conveniently improves the ampoule’s readability and reduces the potential for error.

## Discussion

Improving patient safety is an increasing priority for surgeons and hospitals, as sentinel events can be catastrophic for patients, caregivers, and institutions. The World Health Organization (WHO) is making a great effort to put updated guidelines to ensure patient safety in the OR.

The administration of a wrong medication is a known source of error. This has resulted in devastating consequences to the patients and has affected the health care professionals and hospitals.

One of the reasons for this common and potentially fatal error is using the wrong medication because of similar ampoule shapes and colors. A wide range of plastic, embossed ampoules, including those containing local anesthetics, potassium, and saline, resulting in poor contrast between the typeface and background, presenting a challenge for accurate and easy identification.

In the OR, the surgeons and medical staff are responsible for ensuring the administration of the correct medication to the patients. However, similar ampoules and difficult-to-read typefaces increase the risk of compromising the sterility of the surgical table as well as injury to the personnel. By adding a simple step to help in the differentiation between ampoules and improve the visualization of the script, we could help avoid this common error.

## Conclusions

Safety in the OR is an important issue in any hospital, as it involves the safety of the working personnel facing occupational hazards in the OR as well as the safety of the patients. We find that dipping the clear, plastic, embossed ampoule in readily available povidone-iodine antiseptic solution at the start of the procedure, or when needed, improves contrast and reading and renders the vial sterile. This conveniently improves the ampoule’s readability and reduces the potential for error.
